# Supplementary material for: Are carbon emissions trading and green financial instruments synergistic? -Comprehensive quantitative research based on content analysis
Source: PLoS One. 2024 Mar 7;19(3):e0298601. doi: 10.1371/journal.pone.0298601 (PMC10919601; doi:10.1371/journal.pone.0298601)
Supplement: S1 Data — (ZIP) [file pone.0298601.s001.zip › Data/地级市Z_F工作报告.zip!/╔╜╢1⁄2/▒⌡╓▌╩╨/└┤╫╘╣1⁄2╓┌║┼í╛┬φ┐╦╩2╛▌═°í┐_2014.docx]

各位代表：

　　现在，我代表市人民政府向大会报告政府工作，请予审议，并请市政协各位委员提出意见。

　　一、过去一年的工作

　　2013年是全面贯彻党的十八大精神的开局之年，也是我市经济社会发展稳中求进的一年。面对世界经济复苏乏力，国内经济下行压力加大，资源、环境刚性约束趋紧等新情况，在市委的坚强领导下，全市上下群策群力，积极应对，扎实苦干，开拓进取，各项工作取得了新成效。

　　(一)经济保持稳定增长，整体实力进一步提升。预计地区生产总值实现2100亿元，增长9.5%;固定资产投资完成1530亿元，增长21%;社会消费品零售总额实现657亿元，增长12.9%;规模以上工业增加值增长12%;工业主营业务收入突破7000亿元，增长16%;新增规模以上企业270家;城镇居民人均可支配收入实现28236元，增长9.4%;农民人均纯收入达到11353元，增长13%。金融机构本外币存贷款余额分别实现1927亿元、1692亿元，增长13.5%、12.5%。

　　(二)加快转型升级步伐，质量效益逐步提高。预计高新技术产业实现产值1500亿元，占规模以上工业总产值比重达到24.5%，连续三年被评为“全国科技进步先进市”。预计服务业增加值实现810亿元，占GDP比重提高0.6个百分点。公共财政预算收入实现170.09亿元，增长6.05%，税收占比达到75.4%，可比口径提高2.9个百分点。预计万元GDP能耗下降3.7%，主要污染物排放总量完成全年控制目标任务。滨州经济开发区升级为国家级经济技术开发区，高新区通过省级高新技术产业开发区审定。

　　(三)强化农业基础地位，农村经济加快发展。战胜前旱后涝的自然灾害，粮食总产达到266万吨，实现丰产丰收。林水会战成效显著，农田水利基本建设不断加强，新增和恢复蓄水能力5677万方，新建和提升高标准农田54万亩，新增造林22.9万亩。科技兴农步伐加快，黄河三角洲(滨州)国家农业科技园区基础设施、项目建设、机制创新加快推进。沿黄生态高效现代农业示范区被确定为省级现代农业示范区。“渤海粮仓”工程实现新突破，在中轻度盐碱地上大面积种植小麦获得成功，无棣、北海经济开发区盐碱地改良种植水稻实现丰产。农机大田作业面积不断扩大，机采棉技术逐步推广。大力发展生态健康安全的现代畜禽养殖模式，预计肉蛋奶总产量可达83.4万吨。成功举办中国(滨州)黄河三角洲首届农超对接洽谈会。京博、滨化等一批企业集团投资农业，带动了产业升级，促进了农民致富。

　　(四)把握“两区一圈”战略机遇，重大基础设施建设快速推进。紧紧抓住“黄蓝”两区和省会城市群经济圈发展机遇，争取上级各类专款和转移支付补助70多亿元，增长17%。与省发改委签订了加快推进“两区一圈”开发建设战略合作框架协议，与周边市进行了全面对接。滨州港实现试通航。德龙烟铁路滨州段开始铺轨、阳信火车站变更获批并施工，黄大铁路项目获得国家核准。济滨东高速公路开工建设，无棣至莱州公路滨州段、埕口(鲁冀界)至沾化高速公路、长深高速高青至广饶段前期工作加快推进。大唐滨州2×35万千瓦热电联产项目获得国家发改委正式核准。

　　(五)统筹推进城乡建设，城乡面貌明显变化。围绕中心城区建设，实施了黄河五路综合改造、秦皇河湿地公园等一批重点城建项目，全面完成数字滨州二期工程，顺利通过国家园林城市考核验收。中海航母、国际会展中心等社会关注问题得到积极解决。加快县城改造提升，坚持新城区和老城区结合，开发经营与品质提升结合，高起点规划，高标准建设，打造了无棣古城等一批亮点工程。积极推进示范镇和农村社区建设，10个镇列入“省级示范镇”，预计全市城镇化率达到51%。农村环境综合整治效果显著，市财政投入专项奖补资金4620万元，各县区加大投入，带动社会投资8亿多元，超额完成农村道路硬化、村庄净化、村容美化年度任务，完成1148个村居、2788公里的道路硬化。

　　(六)突出保障改善民生，社会事业全面进步。始终把人民群众共享改革发展成果摆在重要位置。财政投入民生事业148.9亿元，占财政支出的59.2%。承诺的16项民生实事全部兑现。新增城镇就业7.2万人，农村劳动力转移就业16.4万人。中小学校舍安全工程和学前教育三年行动计划如期完成，平安校车实现全覆盖，滨州职业学院以优秀等级通过国家骨干高职院校验收。居民养老保险基础养老金标准提高到每人每月70元。农村低保标准提高到每人每年平均2520元，城市低保标准提高到每人每月平均430元。启动新农合20类大病保险工作，探索实施了乡镇卫生院住院“起付线以上全报销”制度，建成467所村级标准化卫生室，全面完成省下达的年度人口和计划生育责任目标任务。圆满完成“十艺节”工作任务，荣获组织奖和三项群星奖。成功创建“中国书法城”。在第12届全运会上取得4金、6银、4铜的好成绩，魏桥创业乒乓球俱乐部成功晋级中国乒超联赛。开工保障性住房6259套、棚户区改造3595户，超额完成省下达任务。社会大局持续和谐稳定，被评为“全国社会管理综合治理优秀市”。

　　(七)坚持改革开放，发展活力日益增强。扎实推进行政审批“三集中、两到位”，市级行政许可项目减少157项，成为全省审批事项最少的市之一。健全完善公共资源交易制度，节支增收5.1亿元，荣获“2013全国公共采购先进单位”称号。调整市县财政体制，启动“营改增”试点。实施了食品药品监管体制改革。试行了海域使用权“直通车”制度。5支企业债券融资54亿元，发行量、融资规模均居全省第二位。滨州港一类开放口岸申报工作全面启动。鲁中运达保税物流中心获批设立。预计进出口总额实现80亿美元、增长27%，其中出口35亿美元、增长23.7%，进口45亿美元、增长30%。

　　(八)严格依法行政，政府自身建设不断加强。自觉接受市人大及其常委会法律监督和政协民主监督，虚心听取各民主党派、工商联和社会各界意见。分别办理人大代表建议和政协提案77件、267件，答复率均为100%。开展“作风改进年”活动，不断加大行政问责、效能监察和审计监管力度。严格执行中央八项规定，厉行勤俭节约反对铺张浪费，认真开展公车私用和“庸懒散”专项治理，压减“三公”经费等一般性支出近亿元。司法、民宗、广电、外侨、统计、物价、对台、史志、地震、气象、水文、海事、人防、油区管理、国防动员和妇女儿童、慈善、残疾人等事业也取得了新成绩。

　　各位代表，过去的一年，在形势复杂、任务繁重的情况下，全市经济社会实现了新发展，取得了新成就。这得益于省委、省政府的高度重视、亲切关怀，得益于市委的坚强领导、科学决策，得益于市人大、市政协、社会各界的有力监督、大力支持，得益于全市人民的团结奋斗、拼搏实干，得益于历届班子打下的坚实基础。在此，我代表市人民政府，向勤劳智慧的全市人民，向各级人大代表、政协委员、各民主党派、工商联、各人民团体和各界人士，向驻滨部队、武警官兵、公安干警，向国家部委和省直机关驻滨单位，向所有关心、支持、参与滨州改革发展的海内外朋友，表示衷心的感谢并致以崇高的敬意!

　　在总结成绩的同时，我们也清醒地看到，经济社会发展还存在一些困难和问题：一是经济下行压力依然很大，企业发展面临市场需求不旺、产品价格下降、生产成本上升等问题，对经济运行带来不利影响。二是转方式调结构任务重，推进的力度不够，企业创新能力不足，生态环境建设需要高度关注。三是财政增收基础不够牢固，搞好后续财源建设、优化收入结构仍有大量工作要做，收入质量有待进一步提高。四是面对发展中的一些突出问题，集中研究、攻关突破不够，破解办法不多，安全生产出现问题，教训深刻。五是政府自身建设需要进一步加强，依法行政的自觉性、能力、水平有待提高，干部作风、廉政建设还存有一些问题，营商环境仍需优化等。对此，我们一定高度重视，采取有效措施不断加以解决。

　　二、2014年工作安排

　　今年是深入贯彻党的十八届三中全会精神、全面深化改革的第一年，是实施“十二五”规划的关键一年，是开创滨州科学发展、追赶超越新局面的重要一年。我们既要看到困难和挑战，更要看到优势和机遇，坚定信心，奋进有为，努力实现经济社会发展的新跨越。

　　今年政府工作的总体要求是：全面贯彻党的十八大和十八届二中、三中全会精神，坚持稳中求进工作总基调，牢牢把握加快“两区一圈”建设机遇，落实“六个更加注重”发展理念，突出优化结构，提升效益;突出项目建设，增强后劲;突出以人为本，关注民生;突出环境保护，生态优先;突出改革创新，激发活力，攻坚克难，扎实苦干，努力实现“加快科学发展，增进民生福祉”的目标。

　　今年经济社会发展的主要预期目标是：地区生产总值增长9.5%左右，公共财政预算收入增长10%左右，规模以上工业增加值增长11%左右，固定资产投资增长18%左右，进出口总额增长8%左右，社会消费品零售总额增长12%左右，城镇居民人均可支配收入增长10%左右，农民人均纯收入增长11%左右，人口自然增长率控制在9.4%。以内，全面完成省下达的年度节能减排任务。

　　围绕上述要求和目标，重点做好七个方面的工作：

　　(一)加快转变发展方式，提升发展质量效益。今后几年是滨州发展的关键时期，既面临追赶超越与科学发展的双重任务，也面临扩大总量与提升质量的双重压力。要切实转变观念，坚定不移地调好三次产业结构，调强各产业竞争力，调优财政收入质量，真正把经济发展的立足点转到提高质量效益上来。工业是实体经济的主体，也是转方式调结构的重点。一是抓好现有企业的技术改造。把企业技术改造作为转方式调结构、推动科技进步、加快产业升级的重要抓手，组织实施分行业转型升级行动计划，鼓励支持企业引进新技术、新装备，推动冶金、化工、纺织、机械制造、粮油果蔬等传统产业升级，拉长产业链条，淘汰落后产能，提高产品质量档次，提升产业竞争力。围绕技术改造，加大投资力度，提高技改投入在投资中所占比重，及时兑现财政贴息、税收优惠、信贷支持等各项扶持政策。二是积极发展新兴产业。围绕国家、省鼓励发展的新医药、新能源、节能环保、信息技术、海洋开发和高端装备制造等新兴产业，鼓励企业上新创新，积极引导与国内外联合协作，千方百计加大投资，努力形成新的产业集群，促进区域经济结构优化，培植新的增长点。特别是各开发区要加大新兴产业招商引资力度，加快项目建设进度，在提升产业层次、优化产业结构上做贡献。三是大力抓好科技创新。鼓励自主创新，突出企业主体地位，加快平台载体建设，重点支持西王集团等企业争创国家级技术研发中心。鼓励联合创新，引导企业与大院大所进行产学研合作。鼓励引进创新，支持帮助企业通过直接购买、合资合作等方式，收购国外企业、科研院所，推动企业借力发展。鼓励管理创新，组织企业开展管理提升、成本比对、品牌建设等活动，创新商业模式，拓展营销渠道。进一步解放思想，注重高层次人才培养引进，兑现激励政策，努力营造鼓励创新、宽容失败的创新文化环境。四是积极招商引资。国外先进企业有资金、技术、管理、市场等优势，对外开放、招商引资不仅可以解决资金问题，而且是提高区域竞争力的重要战略。要发挥产业基础和资源优势，着眼于主导产业的建链、补链、强链，力争在引进世界500强、央企等大项目上实现新突破。扩大对外贸易，提高外资利用水平，为企业走出去搞好支持服务。要加大支持力度，明确目标责任，严格考核奖惩，把招商引资任务落到实处。五是推进服务业跨越发展。科学规划布局，加强载体建设，积极发展城市综合体、商业街区。加快发展生产性服务业，引导工业企业进行副业剥离，支持发展科技研发、金融证券、信息咨询、中介评估、服务外包等专业公司。大力发展生活性服务业，支持发展医疗健康、教育培训、社区服务等新兴业态。积极发展文化产业，推进旅游开发，抓好特色旅游乡镇建设。支持非公有制经济发展，落实优惠政策，鼓励创业创新，努力提高服务业占经济总量的比重。(二)突出重点工程、重点项目建设，增强发展后劲。紧紧抓住“两区一圈”区域发展战略机遇和科学发展带来的产业重组变化机会，主动出击，积极作为，下决心抓好一批重点工程、重点项目。一是全力推进工业重点项目建设。引导各级把主要精力向工业集中，把优惠政策向工业倾斜，把优质资源向工业汇集，集中抓好一批投资5000万元以上的项目，完成年度投资565亿元。鼓励支持魏桥、西王、滨化、京博等纳税过亿元的重点企业提升发展，增强辐射带动作用。突出抓好大唐滨州热电联产、中海沥青专用输油管线、滨化搬迁等项目建设。加强项目调度，健全市级领导“包保”责任制，协调解决困难和问题，保证项目按时推进，为今后经济发展打下坚实基础。二是全力推进重点区域建设。突出北部沿海经济增长极地位，坚持“两年苦干、三年成形、五年崛起北部产业新城”的目标，统筹区域规划，创新体制机制，加快经济发展战略重点向北部沿海转移，全力打造轻质高强新材料、石油化工等千亿级和一批百亿级产业集群，努力把盐碱荒地打造成“黄金宝地”。发挥各类开发区、园区在承接产业转移、壮大工业项目上的主体作用，突出做好基础设施、项目建设、招商引资、科技创新四项重点，赋予更加灵活的管理体制，努力提高财政贡献率。邹平经济技术开发区、滨州经济技术开发区、高新技术产业开发区要强化责任意识，率先发展，成为增强地区实力的排头兵、领头雁。健全完善省级以上开发区考核评价体系，实行末位“黄牌”警告制度，促进各类开发区加快发展。三是全力推进重大基础设施建设。组织一批事关滨州长远发展的重大基础设施项目，加快实现基础设施网络与“两区一圈”城市的全面对接、互联互通。打造省会城市群经济圈出海口：搞好论证规划，加快滨州港深水航道、防波堤及配套工程建设，推进滨州港一类开放口岸申报工作;统筹全市特殊监管区域建设，确保鲁中运达保税物流中心封关运行。打造鲁冀沿海公路交通枢纽：加快济滨东高速公路建设，开工埕口(鲁冀界)至沾化高速，加快推进长深高速高青至广饶段、无棣至莱州公路滨州段的前期工作;启动建设北部沿海连接黄骅港的疏港路。加大铁路建设力度：完成德龙烟铁路铺轨及滨州、阳信站场建设，确保年底通车运营;开工建设黄大、寿平铁路，尽快启动滨港铁路二期项目建设，努力打通北接黄骅港、西连京沪铁路、南通胶济铁路的运输动脉。

　　(三)积极发展现代农业，加快农民增收致富步伐。一是切实抓好粮食生产。着眼于“旱能浇、涝能排”，集中力量抓好灌排工程，对田间低洼地带清淤疏浚，推进农业综合开发高标准农田建设，逐步改善农业生产条件。搞好大田农机耕作，实施深松整地、保护性耕作、秸秆还田等措施，恢复并提升地力。实施好高产创建工程，稳定粮食面积、主攻单产提高，推进县、乡整建制高产创建，为全国、全省粮食安全做出贡献。二是深入推进“林水会战”。实施“林水会战”第二个五年计划，抓好骨干道路高标准绿化提升、路域水系治理、平原水库建设等重点工程，强化工作指导，兑现奖补政策。完成黄河防洪工程建设任务。三是加快科技兴农步伐。推进黄河三角洲(滨州)国家农业科技园区建设，重点抓好高科技项目引进，加速建设“中以科技合作示范园”、中科院地理所滨州试验站等重点项目。加强省、市级现代农业示范区建设，发挥好博兴国丰、惠民鑫诚等现代农业园区的示范带动作用，形成现代高效农业样板。推进“渤海粮仓”工程，提高政策支持和服务支撑水平，逐步实现盐碱地上小麦、玉米、水稻种植的规模化。围绕盐碱地开发使用，抓好耐盐碱粮食、林木、畜草良种的开发研究，搞好试种试验。提高畜牧养殖加工科技水平，大力发展现代渔业。发展壮大林果产业，强化冬枣疏密间伐、绿色控害等果品优质高效技术指导，创好品牌，提高效益。抓好基层农技服务组织建设，培养壮大农村实用人才队伍，引领群众走上科技致富道路。四是扶持壮大农业龙头企业。围绕农业产业化、服务社会化、种植订单化、土地集约化、经营市场化，采取财政贴息、金融扶持、股份合作等方式，大力扶持农业龙头企业，特别是扶持从田间到超市、从乡村到国际市场的龙头企业发展壮大，鼓励工商企业投资农业开发建设，力争市级以上农业龙头企业发展到350家。五是稳步推进农村改革。坚持和完善农村基本经营制度，积极培育新型经营主体，鼓励发展家庭农场，力争农民合作社覆盖到每一个行政村。在落实农村土地集体所有权的基础上，稳定农户承包权、放活土地经营权，实现农民土地承包权和经营权分置并行。加快推进农村土地经营权确权颁证工作，加强土地流转管理服务，引导发展多种形式的适度规模经营。全面落实各种惠农政策，让农业更有效益，努力实现农民增收目标。

　　(四)深化改革创新，激发社会发展活力。按照党的十八届三中全会总体部署，积极稳妥地推进各项改革，进一步解放和发展生产力。加快转变政府职能，正确处理政府与市场的关系，切实发挥市场在资源配置中的决定性作用，减少政府对微观经济的直接干预。进一步简政放权，中央、省要求下放的，要毫不保留地放到底。深化行政审批制度改革，精简程序，优化流程，明确时限，提高效率，进一步加大检查监督力度，严格问责制度，优化营商环境。深化公共资源交易制度改革，进一步完善招投标管理机制，努力实现各项交易业务公开、公正、公平。深化财税体制改革，建立健全“全口径”政府预算体系，探索完善市本级财政增长机制，推进市县财政、部门和“三公”经费预决算向社会公开，健全推广政府投资项目代建制;坚持依法治税，深入开展“营改增”试点，积极培植后续财源，优化财税结构，形成财税增长的良性互动。完善国有资产管理体制，加强市属经营性国有资产集中统一监管，努力实现国有资本保值增值。加快社会养老服务体制改革，支持开展各类养老机构连锁经营。加快推进县级公立医院综合改革，逐步推开乡镇卫生院住院“起付线以上全报销”制度。立足滨州实际，做好与中央和省里改革措施的衔接工作，确保科技、教育、文化、卫生等各项改革上下贯通、有条不紊地推进。

　　(五)积极稳妥推进城镇化，推动城乡一体发展。城镇化是扩大内需新的增长点和重要引擎，要按照规划先行、布局合理、基础配套、智能高效的原则，稳妥推进城镇化进程。一是完善提升中心城区。坚持一张蓝图干到底，继续推进“四环五海·生态滨州”建设，使四环成为“城市项链”，五海成为“城市明珠”。坚持“民生城建·城建惠民”，规划建设一批学校、幼儿园、加气站、农贸市场等便民设施;投资2亿元，重点实施群众急需的路桥工程，在新立河上新建黄河四路、十路及长江三路3座桥梁，打通黄河十一路、渤海十七路等10条城区“半截路”，改造拓展城区路网，缓解交通压力;积极协调争取山东高速公路集团支持，彻底解决黄河大桥收费问题;改造城市水系，做好秦皇河北延改造工程;推进秦台河清淤截污，新上沿河小型污水处理厂，搞好流域生态环境综合治理，在取得经验的基础上，着手整治新立河。抓好老城区和城中村改造，提升城市整体水平。强化城市管理，巩固国家园林城市创建成果，争创省级水生态文明城市，创造条件争创国家生态园林城市。加快市民文化中心建设，做好中海航母、国际会展中心招商及后续工作。二是加快推进城镇建设。做好向上争取工作，确保11个全国重点镇申报成功。充分利用扶持政策，扎实做好10个省级示范镇建设。积极推进扩权强镇试点，激发镇域发展活力。重点乡镇、街道、新型社区要做好产业发展规划，培植支撑带动产业，用产业化促进城镇化。要提升城镇的规划建设水平，搞好基础设施配套，不仅楼房住宅多层化，生活质量更要城市化。要推进人的城镇化，做好户籍、社会保障等工作，力争城镇化率达到52%。三是改善农村生活环境。统筹抓好农村电网改造、饮水安全、沼气入户等工程建设。毫不动摇地推进农村环境综合整治，今年再开工完成1859个村、4193公里的道路硬化任务，确保明年全部完成农村“三化”工程，努力建设幸福家园和美丽乡村。

　　(六)积极有效破解难题，提高科学发展能力水平。一是推进土地节约集约利用。依法管好用好土地，坚守红线，严查违法违规用地。改革供地制度，实行用地指标市级集中统筹，引入分配竞争机制，严格项目投资强度、容积率、节地率等指标约束。抓好盘活挖潜，积极推进城乡建设用地增减挂钩，鼓励开展废弃地复垦，对闲置和低效土地实行限期开发，缓解用地指标紧张。深入推进未利用地开发，抓好土地整理项目，实行市级统管，效益市县分成。加快建立“飞地经济”试验区，扩大海域使用权“直通车”制度试点。二是拓宽投融资渠道。积极推进农信社改革，加快发展村镇银行、小额贷款公司、融资性担保公司等地方金融机构。研究利用好新的融资方式，探索发行企业债、市政债、城投债，扩大短期融资券、中期票据、中小企业集合票据融资，积极推动企业上市，鼓励开展境外融资。坚持市内市外、国内国外资金一并争取，贷款、票据、融资、债券等方式综合使用，千方百计解决固定资产投资和流动资金问题。整合有效资产，打造建设好市级经营性融资平台，加大对城市基础设施建设支持力度，采取带资建设等市场化手段，搞好城市经营建设。积极化解政府债务，偿好旧债，合理举债，良性循环，促进发展。三是科学推进生态环境建设。既要“金山银山”，更要绿水青山。科学制定规划，落实环保技术措施，加大监督管控力度，推进节能减排。围绕城市发展和园区建设，规划建设污水处理厂，突出抓好市污水处理厂扩建工程，确保年内投入运行。企业要从自身生存、社会信誉和社会责任的高度，自觉搞好环境保护，除尘、脱硫脱硝等防范治理设施要应装尽装，装则必用，用要监测。对恶意偷排、偷放、偷倒等环境违法犯罪行为，环保、公安部门要密切配合，严惩重罚，严厉打击。

　　(七)全力做好民生事业，维护社会大局和谐稳定。一是优先发展教育事业。坚持立德树人，加强社会主义核心价值体系教育。逐步改革办学体制，落实分级办学责任，提高教学质量。大力发展学前教育，均衡发展义务教育，鼓励普通高中办出特色，加快建设现代职业教育体系，大力支持高等教育发展。全面提高教师队伍整体素质，优化农村教师队伍结构。完成市城区八中、六中、二中新建扩建。二是推动实现更高质量就业。深入实施“万人就业援助计划”，全面做好高校毕业生、农村转移劳动力、城镇困难人员、退役军人等各类群体就业。实现城镇新增就业3.5万人、农村劳动力转移就业5万人，城镇登记失业率控制在3.3%以内。三是加快发展医疗卫生事业。开工建设市中医院和妇幼保健院新院，实施市人民医院、中心医院扩建工程。开展乡镇卫生院规范化建设，新建标准化村卫生室433处。加强基层卫生医疗队伍建设，搞好村医培训，严厉打击非法行医。认真落实人口和计划生育政策，切实稳定低生育水平。四是不断完善社会保障体系。开工建设市社会养老服务中心，新建城市社区老年人日间照料中心55处、农村幸福院100处。居民养老保险基础养老金标准由每人每月70元提高到75元。整合城乡居民基本养老保险和医疗保险制度。全面落实生育保险和城镇职工、居民医疗保险市级统筹。五是繁荣发展文体事业。强化县级图书馆、文化馆、乡镇(街道)综合文化站和农村文化大院、农家书屋建设。加强广播电视和新闻出版管理，严格文化市场执法，深入开展“扫黄打非”。认真备战，做好第23届省运会参赛工作，广泛开展各类群众体育赛事活动，完成1200个村健身广场的建设任务。六是全力维护社会和谐稳定。健全完善立体化的社会治安防控体系和网格化的社会矛盾调处体系，着力化解农民工工资、征地补偿等重点领域的信访矛盾。全面启动“法治滨州”建设，争创“全国法治城市”。加强国防动员和后备力量建设，做好双拥共建，高质量完成征兵工作。改革完善市县乡村食品药品监管体制，实施食品药品放心工程，确保人民群众“舌尖上的安全”。健全并落实校车安全监管长效机制。严格落实安全生产责任制，坚决遏制重特大事故发生。七是做好事关群众切身利益的民生实事。认真谋划，精心实施，集中做好农村公路“三通”工程、秦台河治理等10件民生实事。

　　三、加强政府自身建设

　　打铁还需自身硬。面对新形势、新任务、新要求，我们必须进一步增强创新意识和实干精神，积极作为，敢于担当，锲而不舍，勇于奉献，高标准完成各项任务，切实担负起历史赋予的重任。

　　(一)不断加强学习。深入学习党的十八大和十八届二中、三中全会精神，深入学习习近平总书记一系列重要讲话精神，认真落实省委、省政府一系列重要决策部署，统一思想，凝聚共识，做好结合文章，努力做到实际工作与上级要求同频共振。切实加强对“黄蓝”两区开发、省会城市群经济圈建设等政策的学习研究，科学谋划，加快推进。学习市场经济知识、科技知识和先进地区成功经验，提升思想境界，提高抢抓机遇的能力、创新发展的能力、破解难题的能力和高效落实的能力。　(二)坚持依法行政。面对错综复杂的各种矛盾，必须坚持依法行政。严格执行重大事项集体决策制度，真正做到依法决策、民主决策、科学决策。自觉接受市人大、市政协和社会各界的监督，全面推进政务公开，增加行政透明度。强化对权力运行的制约，加大督查、问责力度，对不作为、乱作为、慢作为等违纪违规行为，严肃责任追究。

　　(三)转变工作作风。牢固树立“功成不必在我”的政绩观，多做打基础、利长远的事。结合党的群众路线教育实践活动，引导广大干部深入基层、深入群众、深入一线，扑下身子解决好群众反映强烈的突出问题，不断凝聚为民服务的“正能量”。坚持把抓落实作为政府工作的永恒主题，强化工作责任，明确年度任务，严格考核奖惩，形成勇于担当、自我加压、扎实苦干、廉洁高效的工作作风，抓铁留痕，踏石留印，确保各项工作高效落实。

　　(四)加强廉政建设。廉洁从政是增强社会公信力的基础，是扩大号召力、倡树施政新风的根基。要严格落实廉政建设各项规定，落实“一岗双责”责任，警钟长鸣，防微杜渐，恪守好权力底线、法律底线。认真落实中央八项规定，坚决反对形式主义、官僚主义、享乐主义和奢靡之风，严格遵守《党政机关厉行节约反对浪费条例》，严肃查处公款送礼、公款吃喝、公车私用、奢侈浪费行为，坚持勤俭办一切事业。严格执行党政机关办公用房标准，及时清理腾退超标办公用房，统筹整合行政办公资源，综合调剂使用，实现闲置资源的公平配置和最大效益。严格财政管理，严肃财经纪律，进一步压减一般性支出。完善领导干部经济责任审计、行政问责制度，强化监督约束，倡树新风正气，牢固树立为民务实清廉的政府形象。

　　各位代表，加快科学发展、追赶超越，是时代赋予的神圣使命，是人民群众的殷切期盼。让我们紧密团结在以习近平同志为总书记的党中央周围，在市委的坚强领导下，脚踏实地，攻坚克难，扎实苦干，开拓创新，为实现“六个翻番”、提前全面建成小康社会的宏伟目标，为建设生态美丽幸福新滨州而努力奋斗!
